# Knowledge, attitudes, practices, and vaccination coverage of medical students toward hepatitis B virus in North Sudan, 2023

**DOI:** 10.7717/peerj.18339

**Published:** 2025-01-02

**Authors:** Osama M.I. Mohamed, Hassan S.H. Mohammedali, Shimaa N.A. Mohamed, Hussein A.S. Ahmed, Alaa A.M. Mohamedsalih, Ola M.A. Abdalgani, Tebian A.A. Mohammedosman, Alaa A.A. Ali, Thuiba A.H. Ali, Othman N.O. Amin, Mohamed A. Issak, Mohamed O. Abdelaziz

**Affiliations:** 1 Sudan Federal Ministry of Health, Dongola, Northern State, Sudan; 2 Sudan Federal Ministry of Health, Karima, Northern State, Sudan; 3 Sudan Federal Ministry of Health, Merowe, Northern State, Sudan; 4 Faculty of Medicine and Health Science, Merowe University of Technology, Merowe, Northern State, Sudan; 5 Faculty of Medicine and Health Science, University of Dongola, Dongola, Northern State, Sudan; 6 Department of Internal Medicine, Faculty of Medicine and Health Science, University of Dongola, Dongola, Northern State, Sudan

**Keywords:** Knowledge, Attitude, Practice, Vaccination, Hepatitis B virus, Medical students, North Sudan

## Abstract

**Background:**

Hepatitis B virus (HBV) is a global health issue, particularly among healthcare personnel, including students because of its occupational exposure pattern. Healthcare Workers and medical students are recommended to have better knowledge, attitudes and good practices and vaccination toward infection control in general and HBV in particular. This study aimed to assess the knowledge, attitudes, and practices of medical students from North Sudan regarding HBV and its vaccination coverage.

**Methods:**

A cross-sectional study was conducted from January to June 2023 among medical students in North Sudan. Data were collected using a structured scale using online Google Forms. Descriptive and comparative analyses were performed using SPSS version 26. Statistical significance was set at *p* ≤ 0.05.

**Results:**

A total of 426 medical students were included in the study. The majority had good knowledge about HBV (86%) and its transmission methods (77%). The majority showed a favorable attitude (77%). The practice score was however very low (15.5%). Older age and advanced academic level were significantly associated with good knowledge (aOR: 3.9; CI 95% [3.69–12.92]; *p* = 0.016, and aOR: 2.6; CI 95% [1.16–6.15]; *p* = 0.020, respectively). Only a third of the students were vaccinated (33.0%), with only a few of them received complete doses (18.6%).

**Conclusion:**

Students from medical colleges in North Sudan had good knowledge and favorable attitudes toward HBV. However, good practice was low. Older age and advanced academic level were significantly associated with knowledge level. Vaccination coverage was also low among the students in this study.

## Introduction

Hepatitis B virus (HBV) is a common viral infection worldwide, including in Sudan. It is a major cause of morbidity and mortality, leading to chronic hepatitis, cirrhosis, and hepatocellular carcinoma (World Health Organization, 2017). According to the World Health Organization (WHO), in 2022, approximately 256 million people worldwide were living with chronic HBV infection, with an estimated 1.2 million new cases and 1.1 million deaths in the same year (World Health Organization, 2021). The primary modes of transmission are mother to child (vertical), sharing contaminated blood products (by blood transfusion, dialysis and needle stick injuries) and unprotected sex (World Health Organization, 2021).

Sudan is highly endemic for HBV, with the most recent data from globalhep.org indicating a prevalence of hepatitis B surface antigen among the general population ranging from 4.41% to 5.51% (Coalition for Global Hepatitis Elimination, 2019). In addition, a previous old study reported a prevalence rate ranging from 6.8% in central Sudan to 26% in South Sudan (currently a separate country) (Kachimanga et al., 2020). However, another recent study reported a prevalence of 15.5% (Mudawi, 2008). The difference in the estimated prevalence rates could be attributed to the improvements in vaccination coverage and healthcare access in recent years, along with advancements in diagnostic techniques and more comprehensive surveillance systems, which could result in a different prevalence rates compared to older studies.

Medical students, as future doctors, are at a high risk of contracting HBV due to the nature of their work (Eltom et al., 2020). Interestingly, the one-year global pooled prevalence of needle stick injuries among healthcare workers was found to be 44.5% (Bouya et al., 2020). In response to this risk, many countries, such as France and Belgium, have developed mandatory vaccination protocols for medical students, whereas other countries, such as the UK and Spain, highly recommend vaccination (De Schryver et al., 2011). The World Health Organization (WHO) recommends HBV vaccination for healthcare workers (World Health Organization, 2023). WHO also recommends that in countries with high hepatitis B surface antigen (HBsAg) seroprevalence (greater than 2% or 5%), all adults should have access to routine screening for HBsAg (World Health Organization, 2023).

Several studies have assessed the knowledge, attitudes, and practices (KAP) of medical students regarding HBV. Studies from Saudi Arabia and India reported overall good knowledge, attitudes, and practices (Alhowaish et al., 2017; Hussain et al., 2016). Regional studies from Ethiopia and Somalia also reported good knowledge and attitudes but poor practice (Abdela et al., 2016; Ali et al., 2023). Locally, a study from the University of Kordofan reported good knowledge and poor vaccination coverage (AbdAlrahman, Humaida & Hammad, 2020).

The childhood HBV immunization program was first introduced in Sudan in 2005 (Northern State Ministry of Health, pers. comm., 2023). However, because most current medical students in the studied universities were born before the start of the program, they were not included in the vaccination program. In addition, no previous studies have been conducted in this study area. Therefore, it is important to assess the awareness of these students toward this infection and its vaccine coverage among them.

This study targeted a large population consisting of five medical colleges in North Sudan, representing more than 7% of the medical colleges in Sudan. The main purpose of this study was to assess the KAP of these students toward HBV and its vaccination coverage among them.

## Materials & Methods

### Study design, setting, and period

The study design used in this study was a multicentre cross-sectional college-based design conducted at five different medical colleges in the Northern and River Nile states of Sudan. The two states make up North Sudan, and there are five main public colleges, which are; University of Dongola, Merowe University of Technology, University of Shendi, Elsheikh Abdallah Elbadri University, and Nile Valley University. The study period was from January to June 2023.

### Study participants

The study targeted medical students officially enrolled in any of the five universities in the two states during the study period. The total study population was 4,502 students. Any medical student enrolled in these universities who provided consent for participation and completed the survey was included in the study.

Sample size was calculated using the formula; sample size (*n*) = *N*/(1 + *N*∗*e*2), considering a confidence level of 95%, a marginal error of 5%, and a proportion of 50%. The calculated sample size was 355 and was increased by 20% to ensure a greater chance of generalization and compensate for low and missing responses. The final sample size was 426. The sampling method was a two-stage sampling process. In the first stage, a convenience sampling technique was employed, where 1,200 students were selected from the total medical students across five colleges. In the second stage, simple random sampling was applied to this subset, from which 426 students were randomly selected for the study.

### Data collection

A structured questionnaire from several previously published studies was used and consisted of 52 items (Hussain et al., 2016; Abdela et al., 2016; AbdAlrahman, Humaida & Hammad, 2020). It consisted of four sections: sociodemographic section (six items); knowledge section (21 items); attitude section (10 items); practice section (10 items); and screening, status, and vaccination coverage (five). To ensure clear understanding, the questionnaire was developed and administered in Arabic, the primary language of the participants. After data collection, the responses were translated into English for analysis. The distribution was in Google Form through Facebook and WhatsApp groups specific to the medical students of the studied universities.

A pilot test was conducted with 10 medical students from each university to assess the understandability and clarity of the instrument for the targeted participants. In addition, we assessed the internal consistency and reliability of the instrument. The result was high reliability with an overall Cronbach’s alpha value of 0.76.

### Data analysis

Data were analyzed using Statistical Package for Social Science (SPSS), version 26. For scoring purposes, any score of 70% or higher was considered good. Chi square, logistic regression and appropriate statistical tests were conducted for assessing the significance associations and their magnitude, considering a *p* value level of ≤0.05.

### Ethical approval

The study was carried out in accordance with the Declaration of Helsinki. Ethical approval was obtained from the Research Ethical Committee of the Northern State Ministry of Health (Approval Number: 2/2023). In addition, written informed consent was obtained from each participant before completing the questionnaire.

## Results

### Sociodemographic data of the students

A total of 426 medical students from four medical colleges participated in the survey, and all provided consent and fully participated (response rate of 100%).The largest proportion of students (144, 35.2%) were under 21 years old,with a median age of 23 years (inter-quartile range (IQR) of 21–24 years). Two-thirds of the participants were females (66.7%), and half of them were from the last three years of their medical studies (52.6%). Most of the students were single (96.7%) and from urban residents (70.4%). Approximately two-thirds of the students had health insurance (63.6%). These sociodemographic details are illustrated in Table 1.

**Table 1:** Socio-demographics of the participating students.

**Characteristics**	**Frequency**	**Percentage (%)**
**Sex**		
Male	143	33.6
Female	283	66.4
Age (years^*^)		
<21 years	150	35.2
21–23 years	140	32.9
>23 years	136	31.9
**Academic Level**		
First 3 years	202	47.4
Last 3 years	224	52.6
**Marital Status**		
Single	412	96.7
Married	14	3.3
**Health Insurance**		
Yes	271	63.6
No	155	36.4
**Residence**		
Rural	126	29.6
Urban	300	70.4

**Notes.**

* Median (IQR) Age: 23 (IQR of 21–24).

### Knowledge of the studied students about HBV

Regarding knowledge, most students had heard about HBV (96%) and knew that it is a transmissible viral infection (93%). They were aware of contaminated needles and surgical tools were the most common mode of transmission among healthcare workers (97%). Students knew about the consequences of HBV, including chronic hepatitis (93%), cirrhosis, and cancer (87%).

However, only half of the students knew that there is no definitive treatment for HBV (57%) and that most of the infected people are asymptomatic (58%), but the majority (83%) were aware of the availability of vaccination.

In general, most students demonstrated good knowledge about HBV, with an overall score of 84%. The knowledge assessment data are shown in Table 2.

**Table 2:** Knowledge of the students.

**Knowledge-Assessing Questions**	**N (%)**	**N (%)**
**Answer Options**	**Yes**	**No**
1. Have you heard about HBV infection?	407 (96.0%)	19 (4.0%)
2. HBV is an infectious viral disease that can be transmitted from one person to another	397 (93.0%)	29 (7.0%)
3. Know about the modes of transmission of HBV infection?	327 (77.0%)	99 (23.0%)
4. Contaminated surgical tools represent one of the most common HBV transmissions among healthcare workers	415 (97.0%)	3.0%)
5. HBV causes acute and chronic hepatitis	396 (93.0%)	30 (7.0%)
6. The majority of people infected with Hepatitis B virus are asymptomatic	249 (58.0%	177 (42.0%)
7. Hepatitis B virus can cause liver cirrhosis	369 (87.0%)	57(13.0%)
8. Hepatitis B virus can cause liver cancer	369 (87.0%)	57 (13.0%)
9. There is definitive cure for Hepatitis B virus	184 (43.0%)	242 (57.0%)
10. There is a vaccination for Hepatitis B virus	355 (83.0%)	71 (17.0%)
**Overall Knowledge Score**		
Good	358 (84.0%)	
Poor	68 (16.0%)	

As illustrated in Table 3, students had a good understanding of the means of HBV transmission, including blood transfusion (96%), contaminated syringes and surgical tools (97%), shaving tools, toothbrushes, and nail cutters (75%), sexual intercourse (73%), tattooing and traditional cautery (71%), and mother-to-child transmission during pregnancy and birth (75%).

**Table 3:** Knowledge of the students toward the transmission modes of HBV.

**Questions**	**N (%)**	**N (%)**
**Answer options**	**Yes**	**No**
1. Transfusing contaminated blood	410 (96.0%)	26 (4.0%)
2. Sneezing and Coughing	32 (31.0%)	394 ((69%)
3. Syringes and other surgical tools	415 (97.0%)	11 (3.0%)
4. Shaving tools, toothbrushes, and nail cutters	319 (75.0%)	107 (25.0%)
5. Hand shaking and hugging	61 (14.0%)	365 (86%)
6. Sexual contact	310 (73.0%)	116 (27.0%)
7. Tattooing, and Kaiy (traditional cautery)	304 (71.0%)	122 (29.0%)
8. Dialysis	278 (65.0%)	148 (35.0%)
9. Sharing dining and drinking tools	144 (34.0%)	282 (66.0%)
10. From mother to child during pregnancy and birth	321 (75.0%)	105 (25.0%)
**Overall Knowledge Score in the HBV Transmission Modes**		
Good	327 (77.0%)	
Poor	99 (23.0%)	

Some misconceptions regarding certain means of transmission were identified, such as thinking that HBV can be transmitted through sharing dining and drinking tools (33%) or sneezing and coughing (31%) and not by dialysis (35%).

Overall, the knowledge of the students about the means of HBV transmission was good (77%).

### Attitude of the studied students toward HBV

In terms of attitudes (Table 4), most students demonstrated a positive attitude towards managing HBV. A majority disagreed with misconceptions about HBV, such as it being treatable with herbal and traditional medicine (77%) or with traditional cautery (86%). Furthermore, 92% of students correctly believed that HBV carriers cannot donate blood, while 87% believed that HBV carriers can pursue education.

**Table 4:** Attitude of the students.

**Statement**	**Frequency**	**Percent (%)**
**Options**	**Agree**	**Disagree**
1. HBV infection can be treated using herbal and traditional medicine	97 (23.0%)	329 (77.0)
2. HBV infection can be treated with Kaiy (traditional cautery)	61 (14.0%)	365 (86.0%)
3. HBV carriers can donate blood	32 (8.0%)	394 (92.0%)
4. HBV carriers can pursue education	369 (87.0%)	57 (13.0%)
5. Using condoms during sexual intercourse prevents HBV transmission	234 55.0%)	192 (45.0%)
6. Medical and health sciences students and healthcare workers should be vaccinated against HBV	401 (94.0%)	25 (6.0%)
7. Pregnant women can receive vaccination against HBV	184 (43.0%)	242 (57.0%)
8. Mothers infected with HBV should not breastfeed	266 (66%)	160 (38.0%)
9. If a medical student gets infected, he/she should seek medical treatment and continue to study while being careful not to transmit the infection	398 (93.0%)	28 (7.0%)
10. Screening for Hepatitis B virus before marriage is important	399 (94.0%)	27 (6.0%)
**Overall Attitude Score**		
Good	329 (77.0%)	
Poor	97 (23.0%)	

Additionally, 94% of students agreed that medical and health science students, as well as healthcare workers, should be vaccinated against HBV. The importance of screening for HBV before marriage was also emphasized by the majority (94%). Similarly, a significant proportion (93%) agreed on the correct steps for managing needle stick injuries.

However, some misconceptions persisted. Notably, 45% of students believed that using condoms during sex does not prevent HBV transmission, 57% thought that pregnant mothers cannot be vaccinated against HBV, and 62% believed that HBV-infected lactating mothers should not breastfeed. Despite these misconceptions, the overall attitude score was favorable, with 77% of students having a positive attitude.

### Practices of the studied students toward HBV

In the practice section (Table 3), only a third of the students reported being vaccinated against HBV (33%), and a small percentage completed the doses (18.6%) and received booster doses (10.0%). Similarly, only about one-fifth of the students had ever been screened for HBV (21.1%), of whom, few of them reported positive (6.7%).

Regarding dealing with needle stick injuries, some students reported incorrect practices, such as not pressing the wound to squeeze out blood (69%), ignoring cleaning the wound with antiseptics (63%) or disregarding receiving HBV post-exposure immuneglobulin prophylaxis (58%). Similarly, one-third of the students reported leaving the hospital and just taking antibiotics without adherance to the correct medical guidance (35%).

Regarding personal tools, the majority reported personalizing shaving tools, toothbrushes, and nail cutters (78%) and asking the barber or hairstylist to use new or their own shaving tools (62%). In addition, approximately half reported shaving and styling their hair at the saloon (53%). As illustrated in Table 5, the overall good practice score was relatively low (15.5%).

**Table 5:** Practice of the students.

**Statement**	**N (%)**	**N (%)**
**Answer Options**	**Yes**	**No**
1. Vaccinated against HBV	140 (33.0%)	286 (67.0%)
2. Ever screened for HBV	90 (21.0%)	336 (78.9%)
3. With stick injury from contaminated needle or surgical tool, I should press the wound to squeeze blood out	132 (31.0%)	294 (69.0%)
4. With stick injury from contaminated needle or surgical tool, I should clean the wound with antiseptics	158 (37.0%)	268 (63.0%)
5. With stick injury from contaminated needle or surgical tool, I should take anti-HBV serum	180 (42.0%)	246 (58.0%)
6. With stick injury from contaminated needle or surgical tool, I should take antibiotics and do nothing	148 (35.0%)	278 (65.0%)
7. I personalize shaving tools, toothbrushes, and nail cutters for personal use only	333 (78.0)	93 (22.0%)
8. I ask the barber or hairstylist to use new or shaving tools or your own shaving	263 (62.0%)	163 (38.0%)
9. I cut and style my hair and beard at the saloon or barbershop	201 (47.0%)	225 (53.0%)
**Overall Practice Score**		
Good	66 (15.5%)	
Poor	360 (84.5%)	

**Notes.**

* The remaining 286 (67.0%) reported either not vaccinated or not sure about it.

### Factors associated with the KAP levels

Older age and advanced academic level were the identified significant factors (*p* = <0.001) for higher knowledge (Table 6). The logistic regression test (Table 7) showed that older students (above 25 years) had 3.9 times better understanding than younger students (aOR: 3.9; CI 95% [3.69–12.92]; *p* = 0.016). It also showed that last 3 years students had 2.6 times more knowledge compared to first 3 years students (aOR: 2.6; CI 95% [1.16–6.15]; *p* = 0.020).

**Table 6:** Correlation of basic characteristics of the students participated.

**Variable**	**Knowledge**			**Attitude**			**Practice**		
**Correlation**	**Good**	**Poor**	*P*-value	**Good**	**Poor**	*P*-value	**Good**	**Poor**	*P*-value
**Sex**									
Male	80.4%	19.6%	0.147	79.0%	21.0%	0.531	81.8%	18.2%	0.276
Female	85.9%	14.1%		76.3%	23.7%		85.9%	14.1%	
**Age**									
<21 years	70.7%	29.3%	<0.001	71.3%	28.7%	0.42	88.7%	11.3%	0.208
21–23 years	87.1%	12.9%		77.1%	22.9%		82.9%	17.1%	
>23 years	95.6%	4.4%		83.8%	16.2%		81.6%	18.4%	
**Academic Level**									
First 3 years	73.3%	26.7%	<0.001	70.8%	29.2%	0.003	87.1%	12.9%	0.156
Last 3 years	93.8%	6.3%		83.0%	17.0%		82.1%	17.9%	
**Marital Status**									
Single	83.7%	16.3%	0.314^*^	77.2%	22.8%	0.903^*^	84.7%	15.3%	0.463^*^
Married	92.9%	7.1%		78.6%	21.4%		78.6%	21.4%	
**Health Insurance**									
Yes	82.3%	17.7%	0.192	76.8%	23.2%	0.756	86.3%	13.7%	0.165
No	87.1%	12.9%		78.1%	21.9%		81.3%	18.7%	
**Residence**									
Rural	80.2%	19.8%	0.157	73.8%	26.2%	0.275	88.1%	11.9%	0.185
Urban	85.7	14.3%		78.7%	21.3%		83.0%	17.0%	

**Notes.**

* Fisher’s Exact Test was used to calculate the *p*-value because more than 20% of the expected cell counts were less than 5.

### HBV screening status & vaccination coverage

Regarding screening, only 21.1% of the students reported being screened for HBV, and 6.7% of those who tested were positive. In addition, approximately one-third of the students (33.0%) reported receiving the hepatitis B vaccine. Among them, 42.1% received the first dose, 39.3% received two doses, and only 18.6% completed the three-dose series. These data are illustrated in Table 8.

**Table 7:** Assessment of factors affecting the knowledge and attitude of the studied students about HBV using logistic regression test, 2023.

**Variable**		**cOR (95% CI)**	*P*-value	**aOR (95% CI)**	*P*-value
**Knowledge**	**Age**				
	<21 years	Reference			
	21–23 years	2.8 (1.53–5.16)	0.001	1.68 (0.82 –3.44)	0.155
	>23 years	8.9 (3.69–21.92)	<0.001^*^	3.95 (1.29–12.02)	0.016
	**Academic Year**	
	First 3 years	Reference			
	Last 3 years	5.47 (2.93–10.22)	< 0.001^*^	2.58 (1.45–4.62)	0.020^*^
**Attitude**	**Age**				
	<21 years	Reference			
	21–23 years	1.35 (0.798–2.30)	0.260	0.96 (0.51–1.82)	0.906
	>23 years	2.08 (1.17–3.71)	0.011^*^	1..24 (0.55 –2.77)	0.601
	**Academic Year**	
	First 3 years	Reference			
	Last 3 years	2.02 (1.27–3.21)	<0.003^*^	1.83 (0.95–3.53)	0.071

**Notes.**

* Statistically significant; cOR, Crude Odd Ration; aOR, adjusted Odd Ratio.

## Discussion

The current study was conducted among medical students from five medical colleges in North Sudan. It aimed to assess their vaccination coverage, knowledge, attitudes, and practices toward HBV, as well as the associated risk factors.

**Table 8:** HBV screening, status & vaccination coverage among participant students.

**Characteristics**	**Frequency**	**Percent**
**HBV Screening**		
Yes	90	21.1
No	336	78.9
HBV Status^*^		
Positive	6	6.7
Negative	84	93.3
**HBV Vaccination Status**		
Vaccinated	140	33.0
Not Vaccinated/not sure	286	67.0
Doses HBV Vaccine Received^**^		
One	59	42.1
Two	55	39.3
Three	26	18.6

**Notes.**

* HBV status is reported only among participants who were screened (*n* = 90).

** The number of HBV vaccine doses received is reported only among those who reported being vaccinated (*n* = 140).

In terms of knowledge, the participants generally demonstrated good knowledge (84%) about HBV. This is lower than the findings from the study from Kordofan (92.9%) and higher than those from Ethiopia (80%) and Somalia (73.7%) (Abdela et al., 2016; Ali et al., 2023; AbdAlrahman, Humaida & Hammad, 2020). However, a study from Jordan reported poor knowledge scores (40%) (Alaridah et al., 2023). This difference in knowledge could be attributed to the fact that the study of Jordan included students from other health science faculties, whereas the current study focused solely on medical colleges. Several other differences could be responsible for the variation in results, including differences in educational curriculum, healthcare infrastructure, and cultural beliefs.

Regarding the modes of HBV transmission, 77% of the students correctly identified them. This finding aligns with the results from the Kordofan study (76%) and lower than the study from India (86.1%). (Hussain et al., 2016; AbdAlrahman, Humaida & Hammad, 2020). Again, this slight variation could be attributed to the difference in educational curriculum, and cultural beliefs.

Participants in the present study demonstrated a favorable attitude (77%) toward HBV, which is almost similar to findings from Ethiopia (83.3%) (Abdela et al., 2016). In contrast, a study from Jordan reported a lower favourable attitude level (40%) (Alaridah et al., 2023). The difference in attitude levels may be due to the composition of the study populations. While our study focused specifically on medical students (sample size 426), the Jordanian study included a much broader population of healthcare students (sample size 2322), potentially capturing more diverse levels of knowledge and exposure, which could influence attitudes toward HBV.

Despite the good levels of knowledge and attitude, the current study revealed an overall poor practice score (15.5%). In contrast, the Jordanian study reported a good practice score (63.3%) (Alaridah et al., 2023). Again, this could be attributed to differences in targeted population and the related educational and cultural factors between the two studies.

Older age and advanced academic level were the only risk factors significantly associated with good knowledge towards HBV, while none were significantly associated with practice. These findings are consistent with those of the study from Jordan (Alaridah et al., 2023).

In terms of screening, only 21.1% of participants in the current study had been screened for HBV, with 6.7% testing positive. This finding is lower with regards to screening, compared to a study from India, where 35.7% of participants were screened for HBV (Thote et al., 2023).

Overall, the vaccination coverage among the studied participants was low (33%), with only 18.6% receiving the full doses. These findings are significantly lower compared to the Indian study, which revealed a vaccination coverage of 46.45%, but with only 11.2% being fully vaccinated (Alaridah et al., 2023). The lower vaccination rate observed in this study may be due to several factors. One potential barrier is the lack of awareness about vaccination centers, as many students reported not knowing where to receive the vaccine. Another significant barrier is the high cost of the vaccine, which is approximately $100 for the full three-dose regimen, making it financially inaccessible for some students. Furthermore, vaccination is not mandatory in the studied universities, which may contribute to lower uptake.

The major strengths of the current study include an adequate sample size and representation from five universities, as well as the use of a reliable instrument. However, there are several limitations, including the self-reporting process and the cross-sectional design of the study, which makes it difficult to establish a clear cause-and-effect relationship. In addition, there were controversies and ambiguity in some statements of the assessment scale which may have impacted the accuracy of the assessed KAP rates.

To improve vaccination coverage, a multifaceted approach could be considered. Increasing awareness of vaccination and its locations through targeted information campaigns within universities would be an important first step. Reducing the financial burden by offering subsidies or free vaccination programs could also significantly enhance uptake. Additionally, universities could integrate HBV vaccination into routine health checks or registration processes for medical students, emphasizing its importance as part of professional training. On the other hand, medical students themselves can play a vital role in promoting HBV vaccination by serving as peer educators and advocates. Their involvement in awareness campaigns or workshops could help influence their peers to understand the risks associated with HBV and the importance of vaccination. Furthermore, collaborations with local health authorities to increase vaccine accessibility on campuses could also be effective.

To improve practice and encourage compliance with preventive measures, the researchers recommend achieving strict behavioural change. This can be achieved through training the students for infection control practices and providing them with practice-oriented sessions, case scenarios and role play-based trainings. The researchers also recommend conducting future studies identifying the common malpractices, and exploring personal beliefs and experiences more deeply.

## Conclusions

The current study demonstrated that medical students in North Sudan possess good knowledge and favorable attitudes toward HBV. However, these positive attributes do not translate into high vaccination coverage or effective preventive practices. Notably, the study found low vaccination rates and screening among participants, highlighting a critical gap between knowledge and practice. Older age and advanced academic levels were associated with better knowledge and attitudes, but this did not necessarily improve vaccination uptake.

To address these gaps, it is crucial to focus on improving the accessibility and affordability of HBV testing and vaccination for students. Implementing cost-reduction strategies, increasing awareness about vaccination centers, and integrating vaccination programs into student health services are essential steps. While qualitative research can provide valuable insights into the barriers faced, immediate action is needed to enhance vaccine coverage and testing availability to bridge the gap between knowledge and practice.

##  Supplemental Information

10.7717/peerj.18339/supp-1 Supplemental Information 1 Data Coded for one-point score for each model option.

10.7717/peerj.18339/supp-2 Supplemental Information 2 Questionnaire

## Additional Information and Declarations

### Data Availability

The following information was supplied regarding data availability: The raw coded data are available in the Supplementary File.
