# Supplementary material for: Knowledge, attitudes, practices, and vaccination coverage of medical students toward hepatitis B virus in North Sudan, 2023
Source: PeerJ. 2025 Jan 2;13:e18339. doi: 10.7717/peerj.18339 (PMC11700487; doi:10.7717/peerj.18339)
Supplement: Supplemental Information 2 [file peerj-13-18339-s002.docx]

**Questionnaire**

**Title of Study:** Knowledge, Attitudes, Practices, and Vaccination Coverage of Medical Students toward Hepatitis B Virus in North Sudan, 2023

**Note:** Answer all of the following questions accurately as much as possible

**Section A: Sociodemographic Information**

1. Sex: [ ] Male [ ] Female
2. Age (in years): ________ [ ] Under 21 [ ] 21-23 [ ] Above 23
3. Academic Level: [ ] First 3 years [ ] Last 3 years
4. Marital Status: [ ] Single [ ] Married
5. Health Insurance: [ ] Yes [ ] No
6. Residence: [ ] Rural [ ] Urban

**Section B: Knowledge about Hepatitis B Virus (HBV)**

1. Have you heard about HBV infection?

[ ] Yes [ ] No

1. HBV is an infectious viral disease that can be transmitted from one person to another.

[ ] Yes [ ] No

1. Check the modes of transmission of HBV infection from the list below:

[ ]Transfusing contaminated blood [ ] Sneezing and Coughing

[ ]Syringes and other surgical tools [ ] Hand shaking and hugging

[ ]Sexual contact [ ] From mother to child during pregnancy and birth

[ ] Dialysis [ ] Sharing dining and drinking tools

[ ]Tattooing, and Kaiy (traditional cautery)

[ ] Shaving tools, toothbrushes, and nail cutters

1. Contaminated surgical tools are a common mode of HBV transmission among healthcare workers.

[ ] Yes [ ] No

1. HBV causes acute and chronic hepatitis.

[ ] Yes [ ] No

1. The majority of people infected with HBV are asymptomatic.

[ ] Yes [ ] No

1. HBV can cause liver cirrhosis.

[ ] Yes [ ] No

1. HBV can cause liver cancer.

[ ] Yes [ ] No

1. There is definitive cure for HBV.

[ ] Yes [ ] No

1. There is a vaccination for HBV.

[ ] Yes [ ] No

**Section C: Attitudes toward HBV**

1. HBV can be treated by herbal and traditional medicine.

[ ] Agree [ ] Disagree

1. HBV can be treated by Kaiy (traditional cautery).

[ ] Agree [ ] Disagree

1. HBV carriers can donate blood.

[ ] Agree [ ] Disagree

1. HBV carriers can pursue education

[ ] Agree [ ] Disagree

1. Using condoms during sexual intercourse prevents HBV transmission.

[ ] Agree [ ] Disagree

1. Medical and health science students and healthcare workers should be vaccinated against HBV.

[ ] Agree [ ] Disagree

1. Screening for HBV is essential before marriage.

[ ] Agree [ ] Disagree

1. If a medical student gets infected, he/she should seek medical treatment and continue to study while being careful not to transmit the infection

[ ] Agree [ ] Disagree

1. Pregnant mothers can be vaccinated against HBV.

[ ] Agree [ ] Disagree

1. HBV-infected lactating mothers should not breastfeed.

[ ] Agree [ ] Disagree

**Section D: Practices toward HBV**

1. Have you been vaccinated against HBV?

[ ] Yes [ ] No

1. If yes, did you receive the full three doses?

[ ] Yes [ ] No

1. Have you ever been screened for HBV?

[ ] Yes [ ] No

1. Do you personalize your shaving tools, toothbrush, and nail cutters?

[ ] Yes [ ] No

1. Do you ask your barber to use new or your own shaving tools?

[ ] Yes [ ] No

**What should you do if you experience needle-stick injury?**

1. I should press the wound to squeeze blood

[ ] Yes [ ] No

1. I should clean the wound with antiseptics.

[ ] Yes [ ] No

1. I should take anti-HBVserum.

[ ] Yes [ ] No

1. I should take antibiotics and do nothing.

[ ] Yes [ ] No

1. I should take antibiotics and do nothing.

[ ] Yes [ ] No

**Section E: Screening and Vaccination Coverage against HBV**

1. Have you been vaccinated against HBV?

[ ] Yes [ ] No

1. If yes, how many doses did you receive?

[ ]One [ ] Two [ ] Three [ ] Booster dose

1. Have you ever been screened for HBV?

[ ] Yes [ ] No

1. If yes, what was the result?

[ ]Positive [ ] Negative

***Thank you for your participation***

**!**
